# Adenosine-induced splenic switch-off on [^15^O]H_2_O PET perfusion for the assessment of vascular vasodilatation

**DOI:** 10.1186/s13550-023-01045-7

**Published:** 2023-11-09

**Authors:** Satu Irene Inkinen, Eero Hippeläinen, Valtteri Uusitalo

**Affiliations:** 1 HUS Diagnostic Center, Clinical Physiology and Nuclear Medicine, Helsinki University and Helsinki University Hospital, Helsinki, Finland; 2 Department of Physics, University of Helsinki, Helsinki, Finland

**Keywords:** Adenosine, coronary artery disease, Myocardial perfusion imaging, Positron emission tomography, Radiowater, Stress testing

## Abstract

**Background:**

Splenic switch-off (SSO) is a marker of adequate adenosine-induced vasodilatation on cardiac magnetic resonance perfusion imaging. We evaluate the feasibility of quantitative assessment of SSO in myocardial positron emission tomography (PET) perfusion imaging using [^15^O]H_2_O.

**Methods:**

Thirty patients underwent [^15^O]H_2_O PET perfusion with adenosine stress. Time-activity curves, as averaged standardized uptake values (*SUV*_*avg*_), were extracted from dynamic PET for spleen and liver. Maximum *SUV*_*avg*_, stress and rest spleen-to-liver ratio (*SLR*), and the splenic activity concentration ratio (*SAR*) were computed. Optimal cut-off values for SSO assessment were estimated from receiver operating characteristics (ROC) curve for maximum *SUV*_*avg*_ and *SLR*. Also, differences between coronary artery disease, myocardial ischemia, beta-blockers, and diabetes were assessed. Data are presented as median [interquartile range].

**Results:**

In concordance with the SSO phenomenon, both the spleen maximum *SUV*_*avg*_ and *SLR* were lower in adenosine stress when compared to rest perfusion (8.1 [6.5, 9.2] versus 16.4 [13.4, 19.0], *p* < 0.001) and (0.81 [0.63, 1.08] versus 1.86 [1.73, 2.06], *p* < 0.001), respectively. During adenosine stress, the SSO effect was most prominent 40–160 s after radiotracer injection. Cut-off values of 12.6 and 1.57 for maximum *SUV*_*avg*_ and *SLR*, respectively, were found based on ROC analysis. No differences in *SAR, SLR*_Rest_*,* or *SLR*_Stress_ were observed in patients with coronary artery disease, myocardial ischemia, or diabetes.

**Conclusions:**

SSO can be quantified from [^15^O]H_2_O PET perfusion and used as a marker for adequate adenosine-induced vasodilatation response. In contrary to other PET perfusion tracers, adenosine-induced SSO is time dependent with [^15^O]H_2_O.

## Background

Positron emission tomography (PET) using [^15^O]H_2_O is a gold standard for quantitative noninvasive imaging of myocardial perfusion and its accuracy for myocardial ischemia has been validated in previous multicenter studies [1–4]. Adequate coronary artery vasodilatation response to adenosine stress is the prerequisite for the successful imaging of myocardial ischemia. Recently, so-called splenic switch-off (SSO) has emerged as a useful imaging marker for adenosine response in cardiac magnetic resonance imaging (CMR) [5, 6]. Adenosine induces splenic vasoconstriction, and the subsequent decrease in spleen blood flow can be seen as a low signal intensity during CMR stress perfusion compared to high signal intensity in the rest perfusion images. Previous CMR studies have shown that SSO is an imaging marker for adenosine-induced myocardial blood flow response [6]. However, the absence of SSO is not necessarily an indicator of failed coronary vasodilatation [6].

The SSO effect has also been previously described in PET perfusion imaging using [^13^N]ammonia and rubidium-82 tracers with adenosine and dipyridamole for stressors [6–8]. Objective imaging marker for successful vasodilator response would be valuable for quantitative PET perfusion as it would allow the differentiation of inadequate vascular adenosine response from balanced ischemia in three-vessel disease or microvascular dysfunction. The technical advantage of PET imaging compared with CMR imaging is that the 3D PET data allow dynamic quantification of spleen, whereas the perfusion slices obtained from CMR are usually not optimized for spleen imaging. In addition, a recent case series reported SSO phenomenon in [^15^O]H_2_O perfusion after adenosine stress [9]. However, the authors only presented the extracardiac finding lacking a dedicated quantitative assessment of the SSO phenomenon.

In our study, we quantitatively assess the clinical utility of SSO as a marker for adequate vascular vasodilator response to adenosine stress on [^15^O]H_2_O PET perfusion imaging. We describe the feasibility and cut-off values of SSO in [^15^O]H_2_O perfusion for further use in clinical practice and research.

## Methods

### Study design

The study population consisted of a retrospective cohort of 30 consecutive patients who underwent [^15^O]H_2_O PET perfusion at rest and adenosine stress at Helsinki University Hospital for evaluation of myocardial ischemia. A history of coronary artery disease, diabetes, hypertension, smoking, and hypercholesterolemia was collected from hospital records. The exclusion criteria were the inability to complete the standard PET perfusion protocol. Written informed consent was obtained from all participants. The study was performed according to the Declaration of Helsinki, and the study protocol was approved by the local ethics committee (HUS/1226/2019) and the Helsinki University Hospital’s institutional research board.

### PET image acquisition

All patients were imaged using a standard clinical [^15^O]H_2_O myocardial PET perfusion protocol. The patients were instructed to abstain from caffeine for 24 h prior to the PET study. Sequential acquisition of helical low-dose computed tomography (CT) for attenuation correction and PET perfusion scans were performed using a digital 4-ring detector time-of-flight PET/CT system (Discovery MI PET/CT, GE Healthcare, Waukesha, WI, USA). Rest perfusion was performed first, and after a 10-min pause for radiotracer clearance stress perfusion was obtained. The target dose was 600 MBq per PET scan (range 416–715 MBq). A standard 6 min adenosine stress was used during the stress perfusion with radiotracer injection 2 min after the initiation of adenosine infusion at 140 µg/kg/min. The scan parameters were identical in both scans. List mode PET data were acquired and reconstructed as dynamic and divided into frames as 14 × 5 s; 3 × 10 s; 3 × 20 s; 4 × 30 s (total time: 4 min 40 s). PET images were reconstructed with the iterative Q.Clear algorithm (GE Healthcare, Waukesha, WI, USA) with 256 × 256 matrix size. The pixel size and slice thickness were set to 2.73 mm and 2.79 mm, respectively.

### PET analysis of SSO and myocardial blood flow

The assessment of SSO was done retrospectively for research purposes only from the time-activity curves (TACs) extracted from the dynamic PET data using Syngo.via (Siemens Healthineers Forchheim, Germany) software’s MM Oncology package. Spherical volumes of interest (VOI) were manually drawn in the liver (mean: 116 cm^3^ [50, 292] cm^3^) and spleen (mean: 11 cm^3^, [6, 25] cm^3^), and then activity concentration (kBq/ml) TAC as average values from the VOI region were exported in a tabular format (*.csv*).

Further processing of the ta TAC values was performed in Python v. 3.8 using custom codes. The average activity concentration values from PET data were transformed into average standardized uptake values (*SUV*_*avg*_) using the administered tracer dose and patient weight. The following parameters were extracted from the *SUV*_*avg*_ TAC: Time to peak *SUV*_avg_ in rest spleen, Maximum *SUV*_*avg*_ value from the spleen TAC, Spleen-to-liver *SUV*_*avg*_ ratio (*SLR*) both during stress and rest (*SLR* = Maximum splenic *SUV*_*avg*_/Maximum liver *SUV*_*avg*_) and the splenic *SUV*_*avg*_ ratio (*SAR* = Maximum splenic activity_Stress_/Maximum splenic activity_Rest_).

No independent ground truth method (such as gadolinium-enhanced CMR in PET-MR) for the SSO assessment was available. However, in our patient population, the spleen maximum *SUV*_*avg*_ and *SLR* values systematically decreased during adenosine stress showing SSO. Only two patients out of 30 had an opposing slight increase in the spleen maximum *SUV*_*avg*_ and *SLR* values in stress compared to rest.

Quantitative global left ventricular myocardial perfusion values were obtained for rest and stress PET scans using Carimas™ (Cardiac Image Analysis System) software package [10]. PET images were read by nuclear medicine physician with more than 10 years of experience with PET perfusion imaging (VU) who established the diagnosis of myocardial ischemia based on the quantitative perfusion data. Previously validated cut-off values for stress myocardial blood flow (MBF) of 2.3 ml/min/g and for myocardial flow reserve (MFR) of 2.5 were used to define the presence of myocardial ischemia [2]. For one patient, the myocardial rest perfusion analysis was omitted due to poor quality of data.

### Statistical analysis

Continuous variables are reported as mean ± standard deviation (SD) and median [interquartile range] for normally distributed and skewed data. Categorical variables are reported as numbers and percentages. Due to sample size (*N* = 30), a nonparametric Wilcoxon signed-rank test was chosen for comparison of stress and rest spleen *SUV*s at different time points and *SLR*s. TACs were used to evaluate the optimal time-points for quantification of the SSO phenomenon. Spleen rest TAC was compared with spleen stress TAC to evaluate the adenosine-induced SSO phenomenon during stress imaging. In addition, boxplot analyses were performed to investigate possible differences in SSO reaction in patients with diabetes, previously known coronary artery disease, or myocardial ischemia at PET perfusion. Boxplots displayed a sample median as horizontal line, a first and third quartiles as boxplot edges, whiskers, which were set to first quantile—1.5 ⋅ interquartile range and third quantile + 1.5 interquartile range, and outliers, i.e., values outside whiskers as circles. The statistical differences between these groups were assessed using Welch’s *t*-test. Correlations were analyzed using Pearson’s correlation.

A receiver characteristics operating curve (ROC) analysis was used to evaluate the optimal cut-off value to differentiate rest perfusion images from the stress perfusion. Area under ROC curve (ROC AUC), sensitivity, and specificity were calculated, and the optimal cut-off point was determined from Youden's index maximum.

The limit for statistically significant differences was set to *p* < 0.001. All statistical analyses were conducted in Python using SciPy (v. 1.10.0) and Pandas (v. 1.5.2), scikit-learn (v.1.2.1) libraries [11].

## Results

The study population consisted of 30 patients who underwent [^15^O]H_2_O PET perfusion imaging. Patient characteristics are shown in Table 1.

**Table 1:** Patient characteristics

Variable	All patients (*N* = 30)
*Demographics*	
Female	9 (30%)
Age (years)	63 ± 11
Body mass index (kg/m^2^)	27.3 ± 4.5
Diabetes	14 (47%)
Hypertension	26 (87%)
Hypercholesterolemia	27 (90%)
Smoking	3 (10%)
Beta-blockers	18 (60%)
Coronary artery disease	11 (37%)
*PET perfusion*	
Ischemia	12 (40%)
Rest MBF (ml/min/g)	1.10 ± 0.25
Stress MBF (ml/min/g)	2.71 ± 0.99
Myocardial flow reserve	2.59 ± 0.90
Stress [^15^O]H_2_O dose (MBq)	601 ± 53
Rest [^15^O]H_2_O dose (MBq)	602 ± 61

Data are presented as mean ± standard deviation or number of patients (%). *MBF* myocardial blood flow

### Quantification of splenic switch-off from PET

**Fig. 1:**
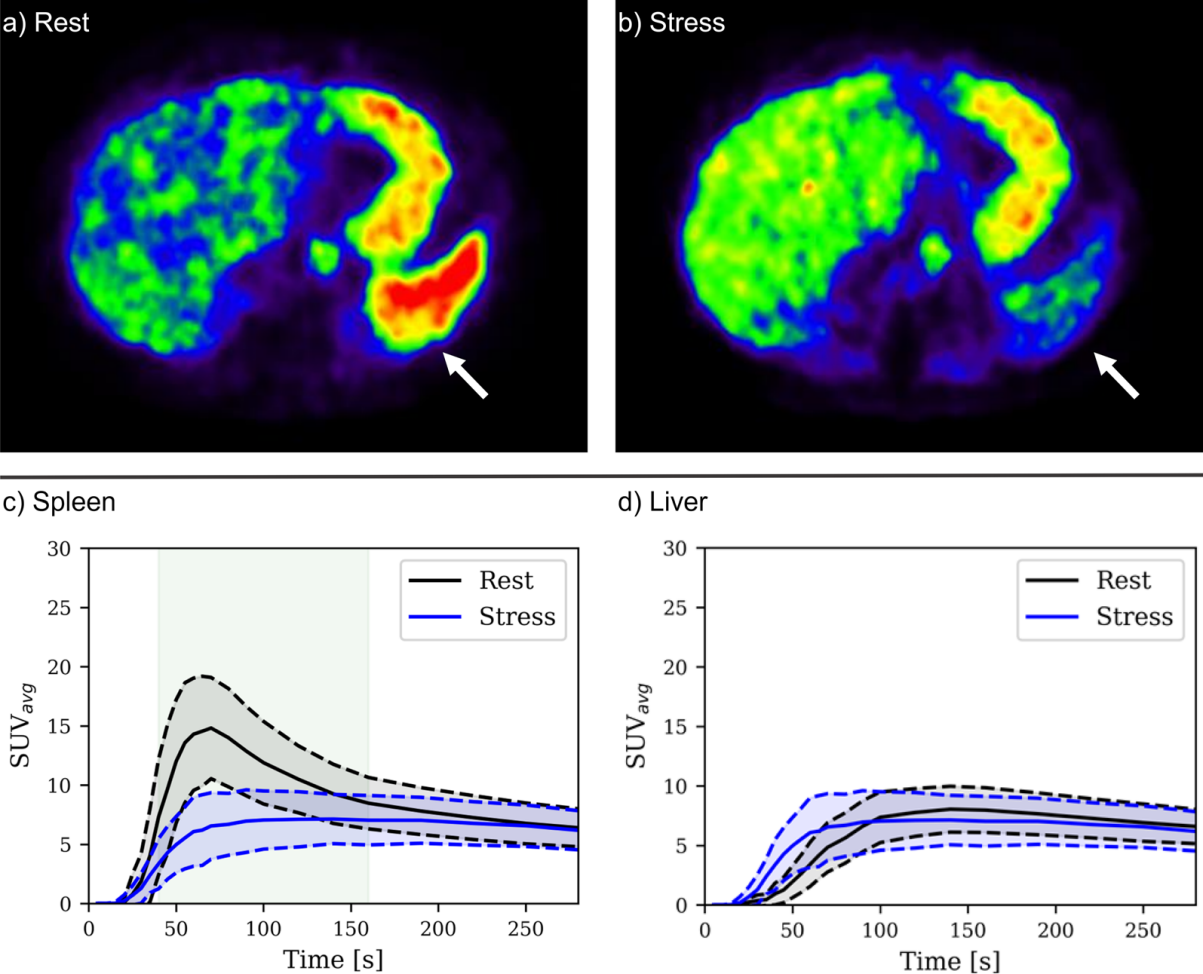
Static 5 s frame reconstruction of rest (**a**) and stress (**b**) perfusion PET scans after 1 min of [^15^O]H_2_O injection (windowing: [0, 140] kBq/ml in both images). The spleen (white arrow) [^15^O]H_2_O perfusion is reduced due to the splenic switch-off phenomenon consistent with a good vascular adenosine stress response. Mean time-activity curves (± standard deviation, dashed line) for rest and adenosine induced stress for spleen (**c**) and liver (**d**). Splenic switch-off effect during stress is shown as decreased [^15^O]H_2_O activity especially at the timepoints starting at 40 s to 160 s (statistically significant difference *p* ≤ 0.001 highlighted in green)

**Fig. 2:**
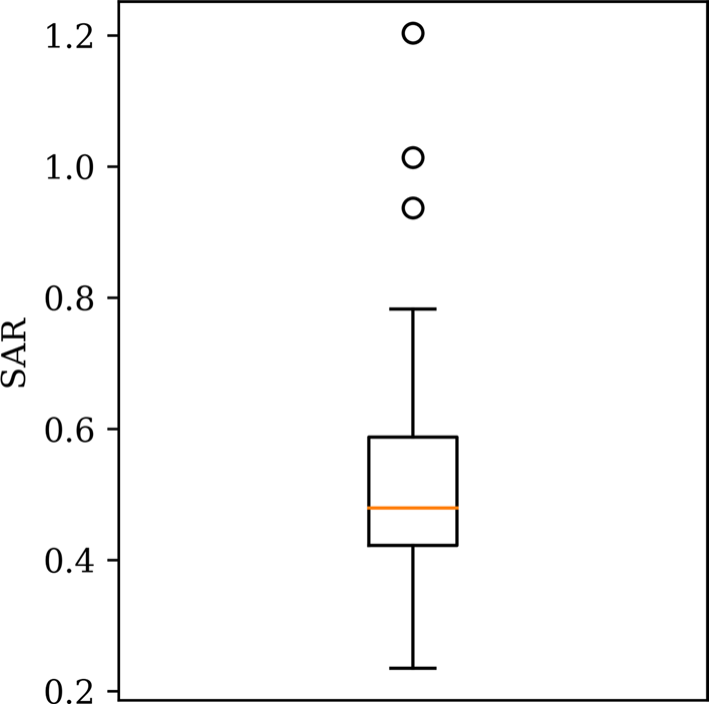
Boxplot of splenic activity ratio (*SAR*) values. Median was 0.48 (orange) and interquartile range (box edges) was [0.42, 0.59]

Differences were observed between spleen rest and stress axial PET reconstructions and TACs (Fig. 1). Dynamic assessment showed that between 40 and 160 s the SSO caused by the adenosine-induced stress statistically significantly decreased *SUV* values (Fig. 1c.). The median time-to-maximum *SUV*_*avg*_ in spleen rest was found to be 65 [55,75] seconds. Both maximum *SUV*_*avg*_ extracted from TAC and *SLR*s were lower in stress compared with rest (maximum *SUV*_*avg*_: 8.1 [6.5, 9.2] vs. 16.4 [13.4, 19.0]; *SLR*: 0.81 [0.63,1.08] vs. 1.86 [1.73, 2.06]) (Fig. 2b, c). The median *SAR* was 0.48 [0.42, 0.59] (Fig. 2).

**Fig. 3:**
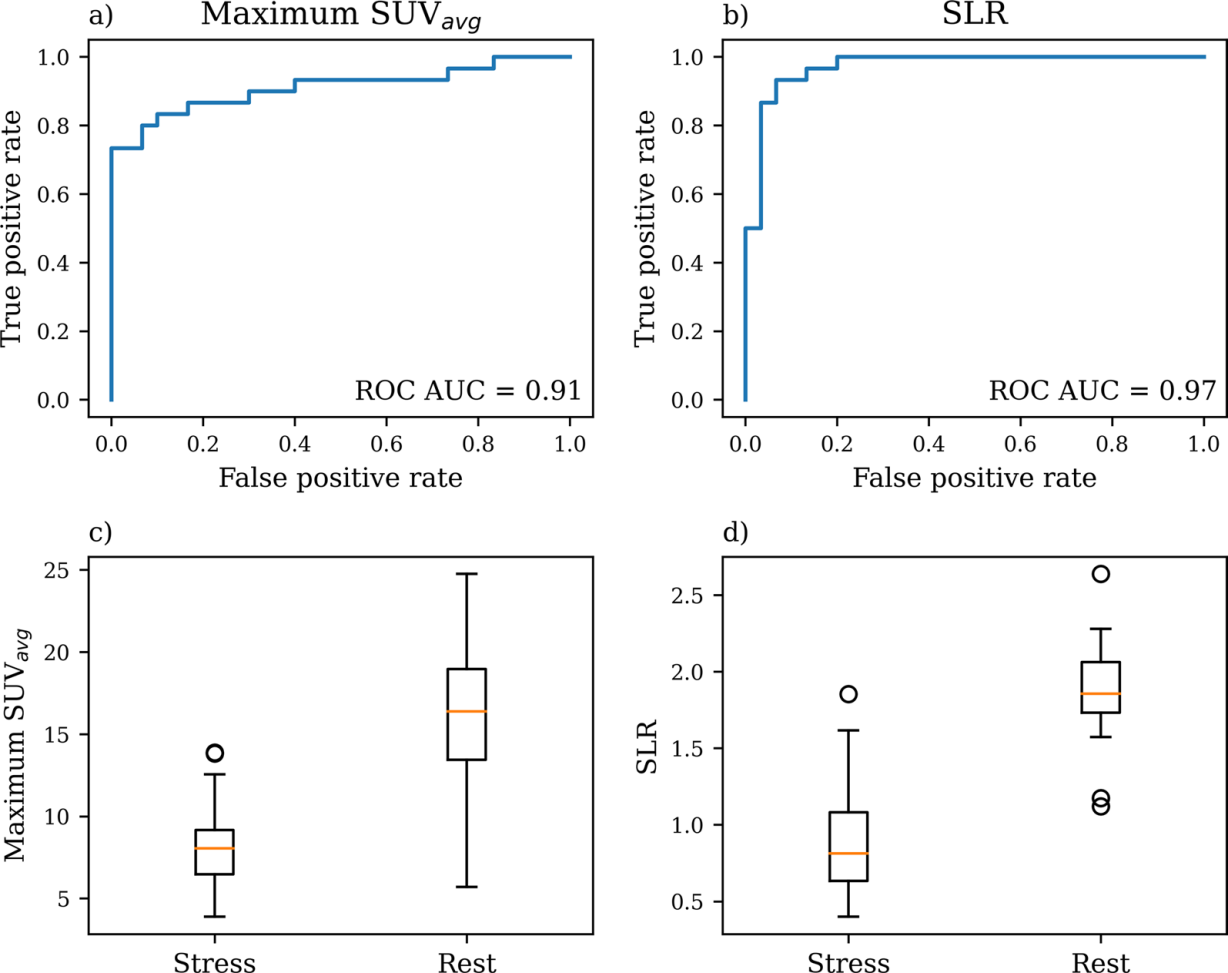
Receiver operating characteristic (ROC) curves for maximum average standardized uptake values (*SUV*_*avg*_) (**a**) and Spleen-to-liver ratio (*SLR*) (**b**) and their area under area under ROC (ROC AUC) values. Boxplots presenting maximum *SUV*_*avg*_ values extracted from spleen time-activity curves (**c**) and *SLR* (**d**). In both cases, there were statistically significant difference between rest and stress groups

When classifying stress and rest based on maximum *SUV*_*avg*_ and *SLR*s, the ROC analysis revealed that the most optimal cut-off values were 12.6 (0.93 specificity and 0.76 sensitivity) and 1.57 (0.93 specificity and 0.9 sensitivity) for maximum *SUV*_*avg*_ and *SLR*, respectively (Fig. 3a, b).

### Association between myocardial [^15^O]H_2_O perfusion and splenic switch-off

**Fig. 4:**
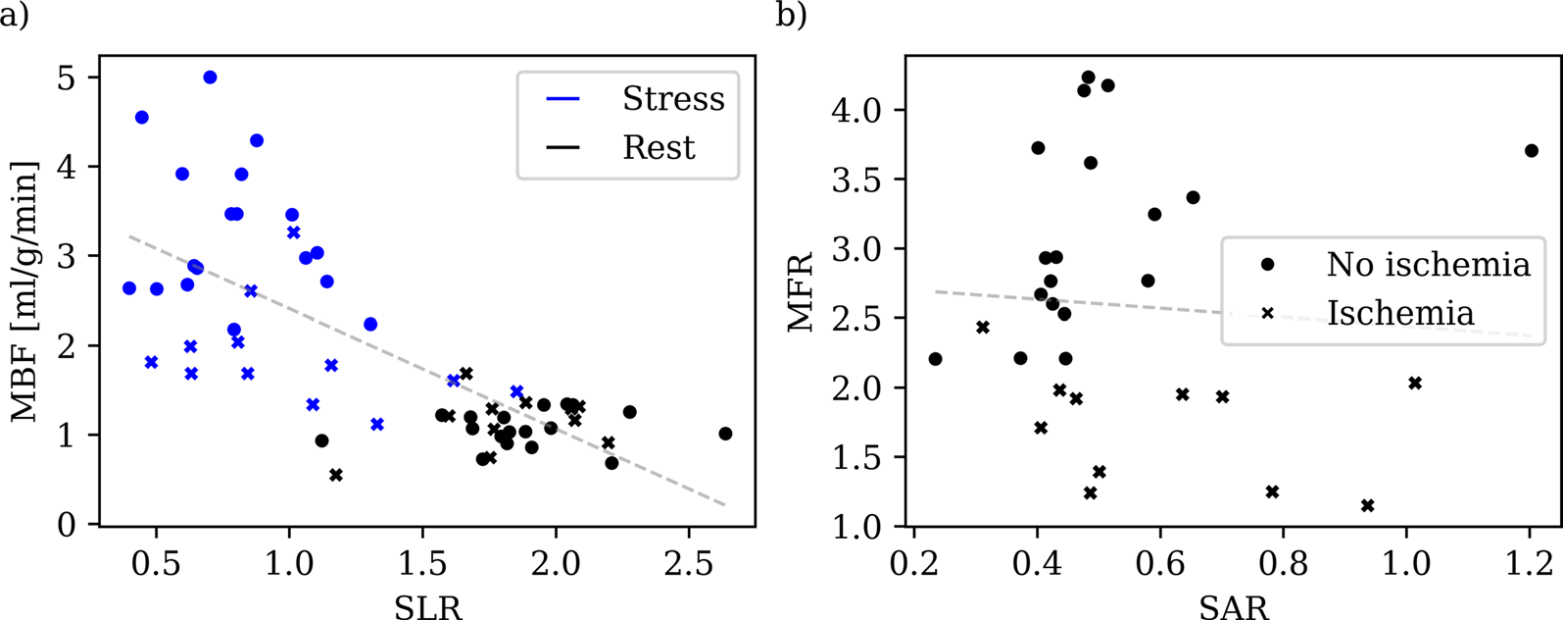
Scatterplots spleen-to-liver ratio (SLR) versus global myocardial blood flow (MBF) (**a**) and spleen activity ratio (SAR) versus myocardial flow reserve (MFR) (**b**). Patients with myocardial ischemia is denoted with × marker in the scatterplots

A strong negative correlation (*r* = − 0.71, *p* < 0.001) was found between *SLR* and global myocardial blood flow (MBF) (Fig. 4a). However, no correlation was found between *SAR* and myocardial flow reserve (MFR) (Fig. 4b)*.* There were 11 (37%) patients with myocardial ischemia of which three patients had global balanced reduction in myocardial perfusion. These three patients received a diagnosis of diffuse atherosclerosis and/or microvascular disease and two of them showed SSO response as a lower maximum *SUV*_*avg*_ and *SLR* in the spleen during stress.

### Risk factors and splenic switch-off

**Fig. 5:**
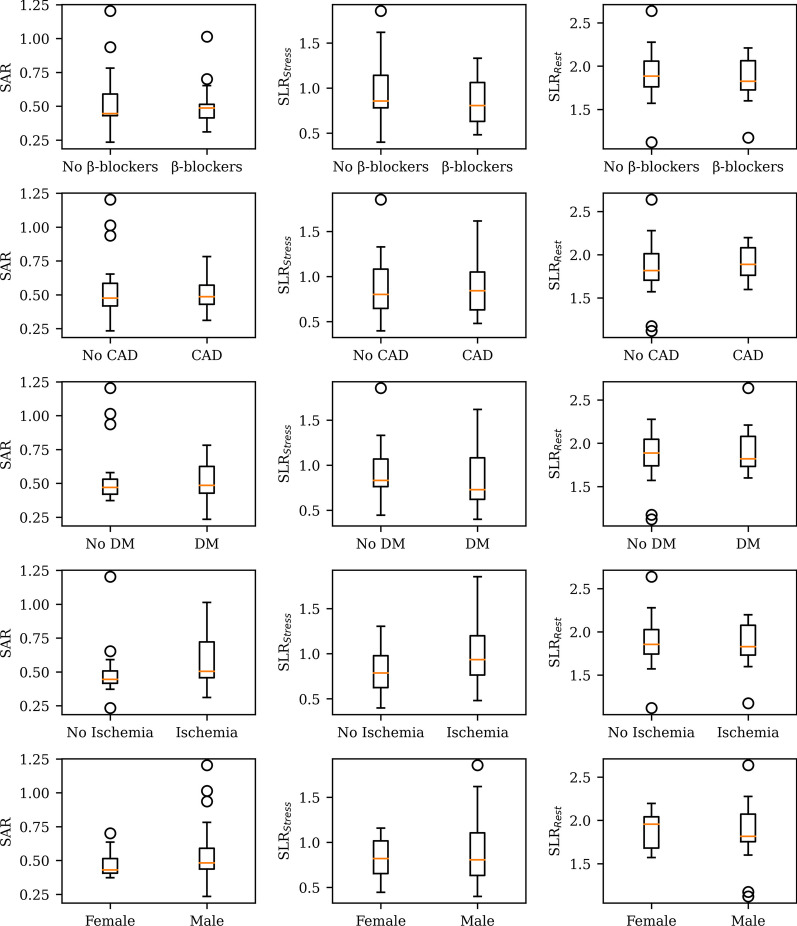
Boxplots showing spleen activity ratios (SAR), rest and stress spleen-to-liver ratios (SLR) for diabetes mellitus (DM), beta-blockers, coronary artery disease (CAD), myocardial ischemia, and gender groups. No statistically significant differences were found between groups

Risk factors should not affect the SSO effect and therefore, additional box plot analysis was conducted (Fig. 5). We found that no statistically significant differences were found between diabetes, beta-blocker use, CAD, gender, and myocardial ischemia in PET patients for *SAR* and *SLR* (Fig. 5).

## Discussion

In this study, we have demonstrated and quantified the adenosine-induced splenic switch-off effect in the [^15^O]H_2_O in stress myocardial perfusion. We show that the [^15^O]H_2_O tracer can be utilized in the SSO assessment, which implies adequate coronary adenosine-induced myocardial blood flow response similar to previous stress CMR and other PET radiotracers studies [5–8]. The visual and quantitative assessment of the SSO phenomenon is most feasible at the perfusion phase of 40–160 s (Fig. 1c). Also, our results indicate that the simpler maximum *SUV*_*avg*_ could also be used in SSO assessment instead of the previously introduced *SLR*. We propose thresholds of 12.6 and 1.57 for stress spleen maximum *SUV*_*avg*_ and *SLR*_Stress_, respectively, indicating a positive SSO effect.

Previous studies have investigated the SSO effect on adenosine stress [^13^N]ammonia PET and dipyridamole stress Rubidium-82 PET perfusion [6–8]. The *SAR* values in our study were similar (0.48 [0.42, 0.59]) to the patients with positive SSO with adenosine stress [^13^N]ammonia PET (0.4 [0.32, 0.45]) [7]. For [^13^N]ammonia and rubidium-82 *SLR*_Stress_ values ≤ 0.92 and 0.71, respectively, were proposed as normal positive splenic response. We observed a quite similar *SLR*_Stress_ values (≤ 1.57) to the previous [^13^N]ammonia threshold. It should be noted that the [^15^O]H_2_O does not accumulate in the liver similar to[^13^N]ammonia and has relatively rapid perfusion as shown in Fig. 1d. Therefore, even though the *SLR*_*Stress*_ values were similar in [^15^O]H_2_O and [^13^N]ammonia, the dynamic kinetic process between the tracers is different which might affect the spleen-to-liver ratio comparisons.

In previous dynamic perfusion PET studies, the kinetics of radiopharmaceuticals have been reported barely. For [^13^N]ammonia, high *SUV* values were reported for the spleen over a whole dynamic rest perfusion PET study (up to 7 min) compared to a stress study [7], indicating positive SSO. We found that the kinetics of [^15^O]H_2_O is more rapid due to tracer wash out (Fig. 1), and timing for SSO assessment is crucial. Based on our spleen *SUV*_*avg*_ assessment, we propose that possible SSO is measured optimally from PET images 60 s after [^15^O]H_2_O injection as this yielded highest statistically significant differences between stress and rest (Fig. 1c). Our study analyzed SSO from a single PET frame (5 to 10 s long) and found that SSO assessment from the dynamic data was feasible. Secondary static reconstruction from the list mode data for the SSO assessment 60 s after injection could be used for visual and quantitative SUV assessment of SSO in clinical practice, as demonstrated in Fig. 1a, b with single 5 s frame.

Our study is unique to previous studies as we describe and validate a simple splenic SUV threshold to quantitatively evaluate the SSO phenomenon from stress-only perfusion images. Previous studies have demonstrated that absolute stress MBF is enough for an accurate evaluation of myocardial ischemia, and rest perfusion can usually be omitted in [^15^O]H_2_O PET perfusion imaging [2, 12]. Spleen SUV quantitation, we describe is a simple and easily standardizable measure for clinical practice and could even be used by technicians after the perfusion imaging as a quality control step to evaluate whether the adenosine stress was successful. Due to the short half-life of [^15^O]H_2_O, stress perfusion can be easily repeated at the same imaging session as necessary with a low additional radiation dose.

The main limitation of this study was the lack of reference standard for PET measured SSO phenomenon. However, we included 30 clinical PET perfusion patients who were screened for caffeine consumption before imaging. Furthermore, comparison with CMR might be problematic due to the superior flow properties of [^15^O]H_2_O compared to gadolinium contrast. Nevertheless, combined PET and magnetic resonance (PET/MR) hybrid imaging would be good modality to cross-validate SSO phenomenon and to evaluate our quantitative PET measures. We did not perform laboratory tests for blood caffeine levels in our study. Interestingly, one previous study showed that SSO imaging failed to detect the effect of caffeine consumption on splenic stress response, which should be explored further in another study using quantitative PET [13]. We observed good vasodilation in the coronary arteries in most cases with quantitative [^15^O]H_2_O PET perfusion. Three patients had balanced ischemia and received diagnosis of microvascular and/or diffuse atherosclerosis, and one of them had poor SSO, which confounded the assessment of coronary adenosine response. A larger patient cohort should be used to validate and assess the diagnostic implications of SSO thresholds derived from our study and assess its impact on patient management. The sample size of our study was small, and we cannot study small differences in quantitative SSO in coronary risk factors such as diabetes or peripheral atherosclerosis possibly affecting the portal blood flow. We were unable to study the associations between hemodynamic parameters, symptom severity, and SSO phenomenon during adenosine stress due to our retrospective study setting, and it should be explored in a further prospective study.

Several factors affect the SUV quantification accuracy. The weight was only asked and was not measured before imaging causing small error in the SUV quantification. The imaging was performed using only a single scanner, and factors such as reconstruction algorithm and parameters, acquisition protocol, and scanner-related physical correction models affect to the SUV quantification. Further prospective validation study should be done prior to clinical adaptation of our quantitative SSO cut-off values to study their accuracy in larger population.

## Conclusions

Splenic switch-off can be observed and quantitatively measured in [^15^O]H_2_O PET perfusion during adenosine stress. [^15^O]H_2_O tracer kinetics were rapid in the spleen, and the statistically significant differences found between rest and stress time-activity curves revealed that the splenic switch-off was most apparent at 40–160 s after tracer injection. In this study, spleen maximum *SUV*_*avg*_ value < 12.6 and spleen-to-liver ratio < 1.57 were found as a simple and clinically useful thresholds to evaluate the adequate vascular response in adenosine stress.

## Data Availability

The data that support the findings of this study are not openly available due to reasons of sensitivity and are available from the corresponding author upon reasonable request.

## Abbreviations

CMR: Cardiac magnetic resonance imaging
CT: Computed tomography
MBF: Myocardial blood flow
MFR: Myocardial flow reserve
PET: Positron emission tomography
ROC: Receiver operating characteristics
ROC AUC: Area under receiver operating characteristics curve
SAR: Splenic activity concentration ratio
SD: Standard deviation
SLR: Spleen-to-liver ratio
SSO: Splenic switch-off
SUV_avg_: Average standardized uptake values
SUV: Standardized uptake value
TAC: Time-activity curves
